# Multilateral Assessment of Anchorage Bond Characteristics in Steel Fibre Reinforced Concrete

**DOI:** 10.3390/polym14071411

**Published:** 2022-03-30

**Authors:** Panagiotis Spyridis, Julia Dreier, Nikolaos Mellios, Lars Walter, Dirk Biermann

**Affiliations:** 1 Chair of Fastenings Engineering, TU Dortmund University, 40476 Dortmund, Germany; connections.bauwesen@tu-dortmund.de (N.M.); lars.walter@tu-dortmund.de (L.W.); 2 Institute of Machining Technology, TU Dortmund University, 40476 Dortmund, Germany; julia.dreier@tu-dortmund.de (J.D.); dirk.biermann@tu-dortmund.de (D.B.)

**Keywords:** bonded anchors, fastenings/anchorages, construction adhesives, retrofit, advanced static three-dimensional numerical analytical approaches

## Abstract

Anchorage to concrete is a recurring application in construction. For such applications, bonded anchors, formed by means of a polymer adhesive injection into a borehole, are a widely used product due to their flexibility in regards to the construction logistics and positioning of the attached element as well as high load capacities. At the same time, fibre-reinforced concrete is the material of choice for many engineering applications where anchors have to be installed. Moreover, the use of steel fibre-reinforced concrete is likely to increase, since it now falls in the scope of the second-generation Eurocode 2 (exp. 2023). Therefore, the condition of the anchor installation borehole—mainly the roughness and grip of its internal surface—is known to play a critical role in the stress transfer from the attached component, through the fastening and into the concrete, and, hence, to the load-bearing performance. At the same time, drilling through the steel fibre reinforcement, along with the accelerated wear of the drilling tools, can in turn influence the borehole’s roughness and the overall installation quality. Furthermore, steel fibre may lead to an additional local stiffening of the concrete where the anchor is installed. These complex elements are discussed herein on the basis of multiple tests on anchors in plain and steel fibre concrete, as well as numerical analyses. The results indicate particular aspects of bonded anchor design and product certification for different polymer-based construction adhesives.

## 1. Introduction

Steel fibre-reinforced concrete (SFRC) is currently the material of choice for a broad range of structures, such as industrial floors (e.g., in manufacturing or processing plants and storage facilities), prefabricated elements, thin shells, segmental and sprayed as well as cast final tunnel linings, special foundations and slabs on grade, watertight and containment structures (immersed structures, silos, and nuclear facilities), and protection and defence structures. Furthermore, its use has also been confirmed for many commonplace concrete component design situations, replacing the entire or a large portion of conventional rebar reinforcements in order to improve their load-bearing behaviour, but also their serviceability and durability characteristics (see, e.g., [1,2,3]). Through the use of fibre reinforcements, concrete’s material characteristics can be improved to align with the acute current and future construction industry objectives, these being a simultaneous increase in the service life of structures and the reduction in their environmental impact, in addition to their resilience to extreme loads and environmental actions. These benefits can be associated, e.g., to a reduction in failure probabilities, improved durability, reduced use of cement and structural steel, thinner and lighter cross-sections, higher ductility, and resistance to impact loads and abrasion [4].

**Figure 1:**
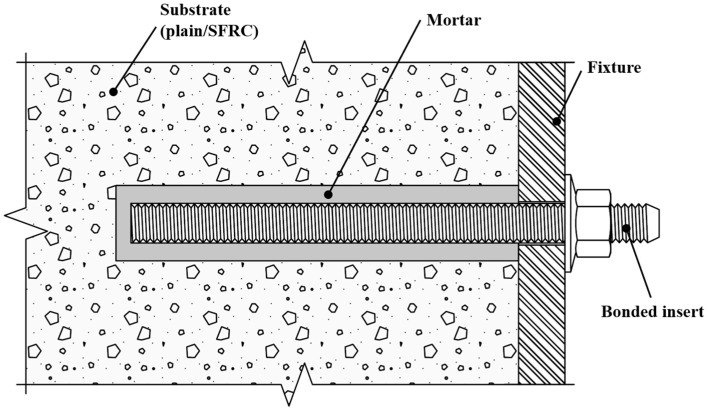
Bonded anchor elements.

Following the extended use of SFRC, modern construction relies heavily on structural connection and assembly technologies. Prominent connection detail relies on post-installed, bonded anchors, which have been the focus of investigations in relation to various substrate materials and loading situations [5,6,7,8,9]. A bonded anchor system comprises a steel insert (often a threaded rod), a bonding agent (mortar/grout), an attached element, and the substrate concrete material, as graphically presented in Figure 1. In most modern heavy-duty systems, the bonding agent is based on a two-component resin injected into a predrilled hole through the use of an appropriate cartridge and dispenser system. Resins can consist of different chemical components, e.g., vinylester, polyacrylate, and polyester and epoxy resins with and without mineral additives, as well as engineered hybrid systems. The installation requires that, after drilling in hardened concrete, the debris and drill dust are thoroughly removed by means of brushing and compressed air, the resin is injected in a liquid state followed by the anchor rod, and the system is left to cure and harden before it is loaded with the attached element and comes to service as a structural component. During the installation and set-up, the processing temperatures and times must be observed, otherwise, the reaction of the resin and hardener would be chemically too strong to process or be disturbed, and would not develop the targeted mechanical properties. The details of the installation, such as drilling equipment, cleaning procedure, borehole geometries, and processing and curing times and temperatures, but also the expected mechanical properties (e.g., bond strength), are defined in detail in the respective product specification document, and they are also noted in the product manual offered by the anchor manufacturer and supplier.

The focus of this paper lies in the influence of steel fibres in concrete with respect to the installation quality and the load-bearing performance of post-installed bonded anchors. To that end, the intersections of the drill hole surface and the steel fibres are qualitatively presented and pull-out tests with varying installation qualities and bonding polymer-based mortars are carried out. An overview of the engineering and design principles of bonded anchors in general and a brief presentation of previous studies for bonded anchors in SFRC are provided, disclosing the state-of-the-art and open questions handled by this study. The set-up and parameters of the tests on bonded anchors in plain concrete and SFRC by means of polymer mortars are reported and their results are discussed. Finally, the findings are summarised with the intention of contributing to practical engineering design and constructability issues. 

### 1.1. State of Knowledge 

#### 1.1.1. Basics of Fastenings Engineering for Bonded Anchors

The design and specification of bonded anchors, as with all post-installed fastenings, are primarily based on the “European Technical Product Specification” (ETPS) for each product. For concrete fastening products, the ETPS is the “European Technical Assessment” (ETA), which was prepared on the basis of a “European Assessment Document” (EAD). The EAD typically prescribes a testing campaign for a product’s assessment. The ETPS also confirms the “Declaration of Performance” through the product manufacturer and allows for the product’s CE marking. The “European Organisation for Technical Assessment” (EOTA) drives this standardisation procedure by endorsing and publishing the assessment principles and by coordinating the organisations carrying out the assessments (“Technical Assessment Bodies”). A similar procedure is followed in the U.S. with the International Code Council (ICC) and the Evaluation Service Reports (ESR). Once the product’s applicability is confirmed by observing the respective specification document, the design can be carried out based on a design norm [10]. For the European regime, this is the Eurocode 2—part 4 “Design of fastenings for use in concrete” [11] and the American counterpart is ACI 318 “Building Code Requirements for Structural Concrete” [12]. The design norm makes use of product-specific mechanical properties which are, in turn, again expected to be found in the product specifications. For bonded anchors, the characteristic resistance against pull-out $N_{Rk,p}^{0}$ is an important feature, which is directly associated with the parameter characteristic bond strength 𝜏_Rk_ as described by Equation (1) (assuming short-term quasistatic but not sustained loading). In Equation (1), *d*, *h_ef_* are the nominal anchor diameter and embedment depth, respectively, in accordance with the uniform bond stress model for bonded anchors in uncracked concrete, as proposed in [13], which simplifies the bond strength to one simple value regardless of the location of the failure surface, i.e., failure at the steel/mortar and mortar/concrete interface, or internal shear failure of the bonding mortar. It has to be also noted that bond stress can be measured by either confined tests (i.e., with support of the tests close to the anchor perimeter) or unconfined tests with a wide support distance. The first case leads to the pure pull-out of the anchor, while the latter typically leads to a mixed-mode failure through the pull-out of the anchor in the lower part of the anchor and concrete cone failure in a part of the anchor closer to the free surface. The test results of a confined pull-out mainly rely on the bond interface to calculate the bond strength per the uniform bond stress model, but they lead to an overestimation of the actual bond strength, since the confinement induces some additional compression stresses in the anchor perimeter. Unconfined tests are rarely used for the bond strength assessment due to the requirements of large concrete member dimensions, (especially for large nominal diameters) and the complicated interpretation of the results, despite the fact that they reflect the real case of anchor tensile function. The combined failure leads to a more realistic pull-out resistance assessment, but the polymer material-specific bond strength evaluation depends on the concrete surface properties, which can be subject to environmental deterioration [14], but also great inherent variability [15], moreover in the case of SFRC [16]. In order to adjust the bond strength from confined tests, it is recommended to multiply it with a reduction coefficient of 0.75 for uncracked concrete (see also [17,18]). Moreover, concrete strength is known to also play a role in the bond strength directly; in [11], a proportionality of an anchor’s bond strength with the square root of the surrounding concrete compressive strength was noted for otherwise identical conditions. Further information on the performance and design of bonded anchors can be found in [19].

(1) $$N_{Rk,p}^{0}=\tau_{Rk}\times \pi \times d\times h_{ef}h_{ef}$$

Focusing on sustained loads, a significant load-performance characteristic of bonded anchors is the point of the “loss of adhesion” on load-displacement curves from short-term quasistatic confined tests. From this point on, the slippage is not controlled by the mortar mechanical and bond properties, and the load-bearing mechanism begins to rely on friction and geometrical irregularities of the borehole. This is typically characterised by an abrupt and significant change in the load-displacement curve. The respective displacement is also set as the limit of long-term displacements to determine the maximum allowable displacements throughout a bonded anchor service life for various assessment approaches [20,21,22], but also in accordance with current design standards [23,24]. 

Another important aspect in the technological development of fastening products is the facilitation of a safe installation since installation defects can substantially decrease the fastening’s structural performance. This is discussed through the example of bonded anchors by [25,26,27], while it is also evident by the fact that [11] recommends an increased resistance partial safety factor for anchorages with a potentially low installation quality. Moreover, many project contractual arrangements require that the installers be trained and certified. The benefits of this certification are discussed in [28,29,30,31,32] and a typical procedure is presented in [33], while standardization institutes provide detailed guidance for the installation of fastenings [34,35]. Notable failures with significant consequences have been attributed to design, specification, and installation deficiencies [36,37].

#### 1.1.2. Previous Research 

Until now, various research studies have examined the performance of anchors in SFRC based on direct pull-out tests on various types of anchors. Ref. [38] reported on axial tests of expansion and composite expansion anchors in normal-strength concrete with corrugated and hooked fibres with a dosage of 50 kg/m^3^ and a steel strength of up to 1050 Mpa. In these investigations, it was found that the expansion anchors could not be installed properly due to fibres crossing the borehole, but further problems with the drilling process have not been reported. Negligible differences in mean resistance values were found between plain and fibre concrete, while anchors in SFRC had a much larger scatter, a phenomenon attributed to the higher porosity of steel fibre concrete as well as the inhomogeneous distribution and orientation of the fibres in the mix. Ref. [39] performed tensile tests on epoxy resin-bonded threaded rods in normal-strength concrete with a steel fibre content of 80 kg/m^3^ and showed no influence on the pull-out capacity of the anchors, but a varying mode of failure was present between unreinforced and fibre-reinforced concrete. Ref. [40] tested different friction-locked anchor types in low-strength concrete with a small fibre content and also found no significant influence of the fibres on the pull-out strength. Ref. [18], on the other hand, carried out unconfined tests on epoxy resin anchors with fibre dosages of up to 70 kg/m^3^ in medium and high-strength concrete and showed that the bond strength increased notably in fibre concrete. In [41], bonded anchors with different mortars were tested in ultra-high-performance concrete (UHPC) with a high strength, high dosage short steel fibre reinforcement. It was found that during hammer drilling, the fibres were not completely separated, presuming additional mechanical bond forces and a more ductile bond failure. Bonded anchor tests in a UHPC are also presented in [42], where a shear failure of the grout was observed, showing a good transfer of mortar stresses into the concrete. Ref. [43] also tested steel and plastic expansion anchors in UHPC and indicated that only plastic anchors failed with pull-out and with a load increase of up to 20% in fibre concrete compared to unreinforced concretes, while the increase between normal and high-strength concrete without fibres was not shown. Extensive reviews of the current state-of-the-art on fastenings in SFRC can be found in [44,45]. Finally, although Refs. [46,47,48] addressed plain concrete, they all confirmed that the roughness and unevenness of the borehole plays a very significant role in the pull-out load. 

#### 1.1.3. Research Gap, Novelty, and Overview of the Study

As seen from the previous sections, research on the anchorage performance in SFRC by means of bonded anchors, particularly on the bond strength, is, thus far, limited, while previous investigations have been predominantly macroscopic without a specific focus on the critical interface of the borehole surface and the polymer adhesive agent. In this paper, the installation quality is specifically investigated as part of the bond performance assessment, and possible adverse influences of the drilling, setting and confinement of bonded anchors by the implementation of steel fibres are specifically discussed with respect to steel fibre reinforcement. The investigated variation parameters include plain and steel fibre concrete, different tool life states of the drill bit, and different polymer construction mortars. Furthermore, borehole surface anomalies and the applied confinement of the anchor by the fibres are discussed. The experimental results on the contribution of the fibres are complemented by three-dimensional FE analyses with a discrete modelling of the fibres in the concrete matrix. 

## 2. Materials

To assess the influence of steel fibres on the bond strength, confined pull-out tests were carried out on unreinforced concrete and steel fibre-reinforced concrete. The fibres used were Dramix 5D BG by NV Bekaert SA, which are filaments of cold-drawn wire with hook-ended shapes, with a length of 60 mm and a diameter of 0.9 mm. The material strength was 2300 Mpa and the rupture strain was 6.0%. For the tests, two different injections resin products were used, namely, (A) methacrylate-based and (B) epoxy-based, both with approval for concrete applications. The metal insert consisted of an M12 threaded rod of high-strength class 12.9. Casting of the specimens was carried out at the laboratory facilities of the TU Dortmund University, Dortmund, Germany. The concrete mix used CEM I 52.5 N Portland Cement and natural aggregates with a maximum size of 16 mm were used. The distribution for all concrete mixes was at 40%, 30%, and 30% for a sieve pass of 0/2, 2/8, and 8/16 mm, respectively. Plain and steel fibre concrete mixes were only varied in that either no fibre reinforcement or steel fibres were added at a dosage of 50 kg/m^3^ during the concrete production in the concrete mixer. All concrete batches had an average compressive strength of 50.9 Mpa and a coefficient of variation of 2%, while fibre-reinforced samples had a consistently higher compressive strength by approximately 1.0 to 1.5 Mpa. The drilling equipment used in all cases included a dry rotary hammer machine and a drill bit with 4 cutting edges, nominal diameter of d_d_ = 14 mm, and a working length of l_d,w_ = 200 mm. The drilling machine applied 850 rpm rotary action and single impact energy of 3.6 Joules. The drill bit was composed of steel, while the cutting tip used a hard metal composite, composed of cobalt-wetted tungsten (wolfram) carbide. 

## 3. Borehole Examinations

### 3.1. High-Level Qualitative Analysis

**Figure 2:**
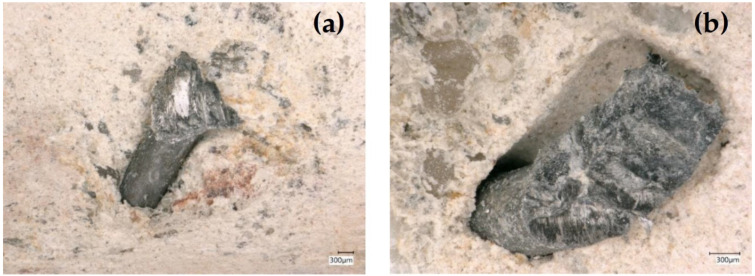
Cross-section deformation (**a**,**b**), local spalling (**b**), and rupture (**a**,**b**) of exposed steel fibres in boreholes.

**Figure 3:**
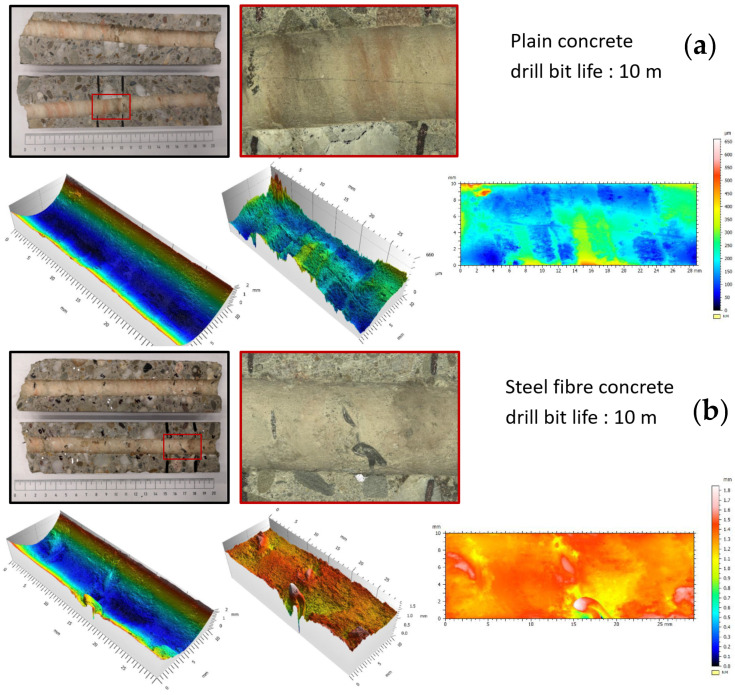
Microscopic assessments of borehole in cored-out specimens in plain concrete (**a**) and SFRC (**b**).

Regarding the behaviour of the steel fibres during the drilling process, it was evident that the fibres led to an increased drilling resistance. Moreover, temperature measurements were taken by means of a laser and contact thermometer, indicating a rise in temperature from 24.0 °C in the neutral condition to an average of 89.2 °C and 99.1 °C after 200 mm long drills in plain and steel fibre concrete, respectively. This 9.9 °C temperature increase also indicated a higher friction stress and respective thermal energy release in SFRC. An optical assessment of the fibres in the borehole indicated a local spalling of the concrete around the exposed fibre and an abrasive separation of the intersected steel fibres in the borehole and a widened end at the fibre separation due to crushing (See Figure 2). The visual assessment of the fibres implied a tendency of the fibres to be pulled out in the rotational direction, but it was unclear whether the fibres were pulled out due to the loss of local bond or whether were plastically elongated. A comparative overall picture of the boreholes after cocentrical core drilling and transverse saw cutting is provided in Figure 3.

### 3.2. Microscopic Roughness Analysis

Quantitative data were retrieved from the internal surface of selected test drills by means of a microscopic investigation. The surface imaging was carried out utilizing the optical 3D coordinate measuring device Alicona Infinite Focus G5 with a lateral and vertical resolution of 0.44 and 10 nm, respectively. The image processing was carried out by means of the specialised surface analysis software μsoft by NanoFocus AG. Due to geometric limitations of the measuring device, only approx. 25 mm of the entire drilling length was recorded per borehole. The examined boreholes corresponded to unreinforced concrete and steel fibre-reinforced concrete.

Following test drillings, cores were taken concentrically to hammer drills and they were cut open along their longitudinal axis for the surface analysis. These are indicatively presented in Figure 3. As seen, boreholes in fibre-reinforced concrete had a generally higher roughness profile, while the intrusion of fibres through the borehole surface led to local peaks in the microscopic scan. Furthermore, this localized damage was accompanied by the spalling of the internal hole surface, which was identified in the microscopic pictures, the 3D coordinate measurement, as well as the profile recording along the longitudinal axis of the borehole. The observation of this spalling with a depth of ~0.5 mm represented a significant difference compared to the surface in nonreinforced concrete. Overall, the roughness of the inner borehole surface in steel fibre concrete was evident. In particular, for boreholes in the averagely worn drill bits, a variance between maximum and minimum profiles was in the range of 0.65 mm for unreinforced concrete and broadens to more than 1.0 mm for fibre-reinforced concrete. 

## 4. Pull-Out Tests at Fully Developed Bond Lengths

**Figure 4:**
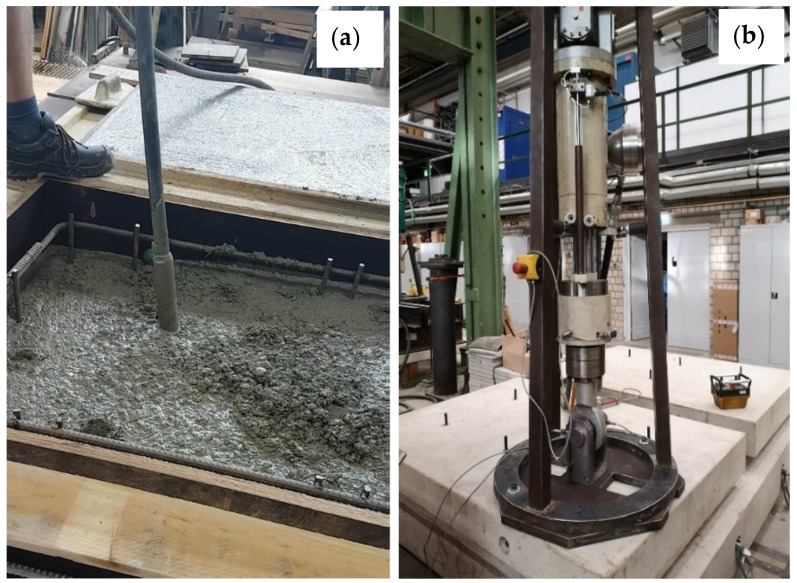
Concreting (**a**) and test arrangements (**b**).

The tests were designed and performed at the laboratory of the TU Dortmund University to exhibit a pull-out type of failure, in which the bond and shear strengths of the injection mortar were the determining factors. The main features of the preparation and execution of tests are provided in Figure 4. The confinement by means of a 40 mm thick steel plate, on which the hydraulic cylinder was located, exerted pressure on the concrete surface in the immediate vicinity of the anchor during the test to prevent a concrete breakout. A PTFE sheet was placed in between to eliminate friction between this plate and the concrete. To pursue a constant stress distribution over the length of the anchor, without stress peaks towards the surface, the upper 20 mm of the rods was wrapped with PTFE tape in several layers, which prevented the mortar from locking into the thread of the anchor rod and also prevented the formation of a load-bearing mortar layer. Hence, the boreholes had a depth of approx. 90 mm and an anchor embedded length of 80 mm, i.e., a bonded length of 60 mm. Any deviation of the anchor axis from the vertical was measured in two perpendicular directions and it always remained below 3° in all cases. 

Apart from the distinction of plain and steel fibre-reinforced specimens, and for each of the two mortar types, the boreholes were distinguished into two groups. Series 1 was cleaned following a procedure of blowing out the dust twice with compressed air, brushing twice with a fitting steel brush, and finally blowing out twice again. Series 2 did not undergo a particular removal of the drill dust from the borehole surface, and it was only ensured that the sediment did not reduce the intended anchoring length; this procedure understandably led to a substandard and potentially defective installation. The drilling was carried out by an existing hammer drill with a four-edge drill bit in all tests. The tests were displacement-controlled with a loading rate between 0.01 and 0.02 mm/s, so as to reach the peak load at approximately 1 to 3 min. The test rig is shown in Figure 2 below. Overall, four tests were carried out for each parameter set, which allowed for a basic statistical interpretation (although product specification tests require a minimum of five tests acc. To EOTA 2018). During testing, test set B2 exhibited an outlier per Grubb’s test. The outlier of test set B2 for test Β2-3 was excluded from the statistical interpretation of the results. The characteristic values were calculated as the fifth percentile, on the assumption of a normal distribution, with a fractile factor of k = 1.83 acc. To Eurocode 0. The test program is given in Table 1. 

### 4.1. Results of Tests with Product A

**Figure 5:**
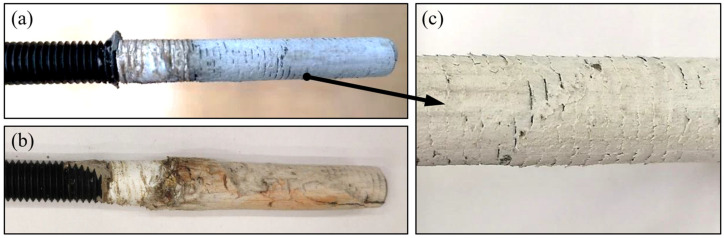
Typical pattern of failure in boreholes with correct preparation (**a**) and substandard cleaning (**b**), and (**c**) detail of failure at the mortar/steel thread interface under good bonding conditions in SFRC—Product A.

**Figure 6:**
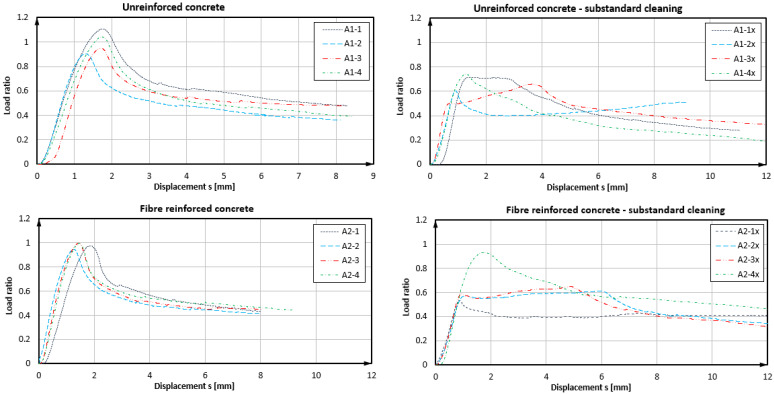
Load displacement curves of confined pull-out tests—Product A.

Indicative failure modes of the anchors are shown in Figure 5 and the load bearing curves achieved through the use of product A are shown in Figure 6 (see also Table 2). The graph developments allowed to qualitatively characterise the failure situation for each bonded anchor [49]. In adequately cleaned boreholes, pull-out tests failed at the interface between the anchor rod and the mortar, due to the shearing of the mortar material. A somewhat different pattern was exhibited for tests in substandard installations with uncleaned boreholes. Tests A1-3x and A2-1x showed a failure between the mortar and borehole wall, whereby the bond strength at the concrete interface was higher than the sliding friction forces during pull-out. A mixed failure could be seen in tests A1-1x, A1-4x, A2-2x, and A2-3x, where the load-bearing throughout the test relied on both friction and the concrete bond strength. Failure in tests A1-2x and A2-4x was likely involved mortar-inherent shearing failure, while the latter appeared to be entirely unaffected by the lack of borehole preparation (good bond strength, despite bad installation quality—marked curve in Figure 3). However, on average, a significant loss of load-bearing capacity was shown in tests with defective boreholes. In normal concrete, the reduction was in the range of 33%, and in FRC, the reduction was 39% (excl. test A2-4x). Considering these differences, as well as the dispersion of load levels and modes of failure, a correlation between the presence of fibres in the mix and the borehole interface characteristics was not statistically validated. Regarding adequately prepared boreholes, the pull-outs in unreinforced concrete exhibited a marginally higher mean value, but also a variation coefficient as compared to the ones in SFRC. By comparison at characteristic levels, it was evident that boreholes with a substandard preparation led to substantially low resistance. In regards to the pull-out tests on anchors with an appropriate installation, it was evident that the anchors in plain concrete exhibited an approximately 12% lower strength than in steel fibre concrete. 

### 4.2. Results of Tests with Product B

**Figure 7:**
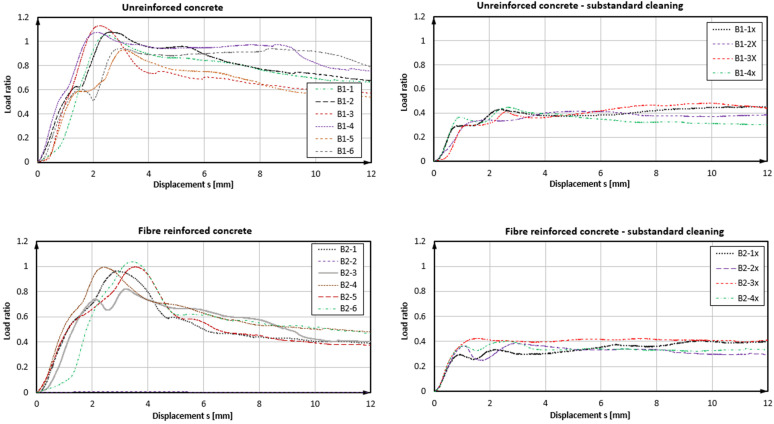
Load displacement curves of confined pull-out tests—Product B.

**Figure 8:**
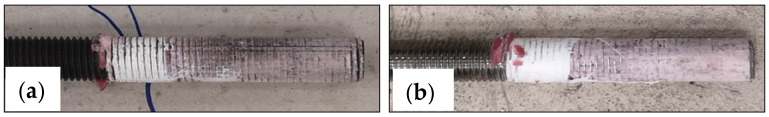
Typical pattern of failure in boreholes with correct preparations (**a**) and substandard cleaning (**b**), both failing with shear mortar failure at thread/mortar interface conditions in SFRC—Product B.

As derived from the load-displacement curves in Figure 7 and exemplarily seen in Figure 8, the bond failure mode was also partially associated with the shearing-off of the bonding mortar at the thread/mortar interface, but also to the loss of the concrete/mortar bond and the load-displacement performance of the anchors, as shown in Figure 7, indicating this behaviour (see also Table 2). It was noted that the further development of the curve beyond 12 mm was virtually flat or slightly decreasing until the complete withdrawal of the anchor from the hole; therefore, it was not included in the graph. In the case of product B and properly prepared boreholes in SFRC, the load-displacement development of bonded anchors reached a peak and then had a distinct decreasing branch in the post-peak region, which implied a development of bond damage, which could, in turn, be attributed to the gradual damage of the mortar material. In plain concrete, the post-failure displacement was flatter, indicating a strong friction component, which could be attributed to a mixed failure surface shared between the mortar/concrete and the mortar/thread interfaces. Moreover, tests in SFRC exhibited a nearly 10% higher bond strength compared to plain concrete and with a much lower variation (2.6% against 7.5%, respectively). Should the outlier be accounted for in test series B2, the mean value would be slightly reduced and the coefficient of variation would reach a similar value to the B1 series. In all cases, the coefficient of variation remained at the quite narrow range for typical fastening test sets. In boreholes without cleaning, the load resistance was dramatically decreased to less than 45% of the nominal value. In these cases, the load-retention development was virtually parallel to the X-axis, denoting a friction component during pull-out after the complete loss of bond in the concrete borehole interface with the mortar. Although displacements at the maximum load were observed for very large displacements, the loss of reliable load-bearing actually occurred at a displacement of 1 mm or less, much lower than in the adequately prepared boreholes. Hence, the results suggested some contribution of steel fibres in the resistance of the concrete–mortar interface and a consequent increase in the pull-out bearing performance of the specific anchors in SFRC for bonded anchor systems with higher strength mortars and a good installation quality. At characteristic levels, boreholes with a substandard preparation again led to particularly low resistance values. In the cases of appropriate installation, the difference between anchors in plan concrete and SFRC was significant, with anchors in SFRC exhibiting approximately 22% higher bond strength design values. A significant element, in this case, was the displacement at the loss of adhesion, which varied between plain and steel fibre concrete, particularly with an adequate installation quality. The average displacement at the loss of adhesion in plain concrete was 1.3 mm with a variation coefficient of 30%, which changed to 1.6 mm and 18% for fibre concrete, respectively.

## 5. Finite Element Analysis of Fibre Contribution

**Figure 9:**
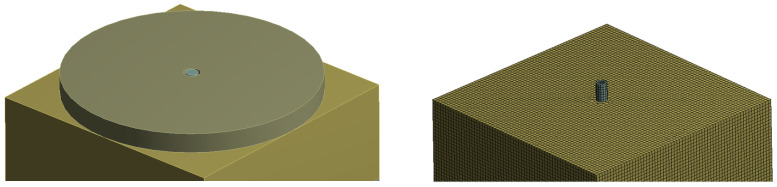
Simulation set-up. Prescribed deformation was applied on the anchor, upwards.

The contribution of fibres in the load-bearing process of bonded anchors was additionally evaluated by means of three-dimensional finite element analyses with discrete modelling and stress measurements of the fibres in the matrix, in line with the modelling approach presented in [50]. The geometry of the models implemented the exact testing configuration discussed in the previous section, as shown in Figure 9. The concrete specimen was supported only in the load axis direction and it was otherwise connected with the steel reaction plate by a free sliding contact interface horizontally. The steel reaction plate was fully supported, and the anchor was loaded at its upper end by means of a controlled displacement. The element used for the solid blocks was SOLID 164, and for the steel fibres, a linear element BEAM 188 based on the Timoshenko theory was implemented, which is a linear two-node beam with six degrees of freedom to each node, and the end nodes kinematically constrained with the solid elements; thus, bearing both axial and transverse loads. The analysis was carried out at the elastic regime and up to a load level corresponding to the loss of adhesion and onset of nonlinear bond behaviour, which was assumed to be 80% of the mean pull-out load, according to the load-displacement graphs of Figure 7. Young’s modulus of elasticity for steel elements was chosen at E = 200 Gpa and the Poisson ratio at v = 0.3. Respectively, concrete was modelled with Young’s modulus of 30 Gpa and a Poisson’s ratio of 0.2. The interface of the support plate and the concrete block mimicked the friction of a PTFE sheet, which was used in the experiments, i.e., with a negligible friction coefficient. The element size for both the solid concrete and linear fibre components was set to 3 mm. 

**Figure 10:**
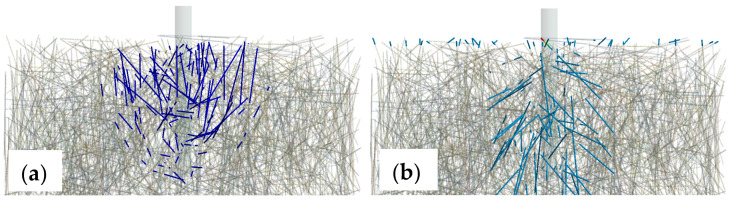
Fibre under compression (**a**) and tension (**b**) exerted by a pull-out load at service level (i.e., prior to failure).

The results indicated that the fibres were activated under the proposed loading conditions both in compression and tension (see Figure 10). The images highlight fibres with compression forces above 5 N, while the maximum compression force reached 78 N. In tension, again, only fibres with a tensile force higher than 5 N were shown. In this case, the maximum tension reaches is 663 N, and only locally in one fibre at the upper boundary. Hence, the contribution of the fibres could be characterised as insignificant regarding the developed load-bearing mechanism. The aggregate numerical value of compression load taken by the fibres in arbitrary directions was 12.9 kN, which indicated an influence in the confinement of the anchor close to the support annular gap of the test configuration, and, hence, possibly an indirect influence particularly for the confined test set-up.

## 6. Radial Stress Assessment

**Figure 11:**
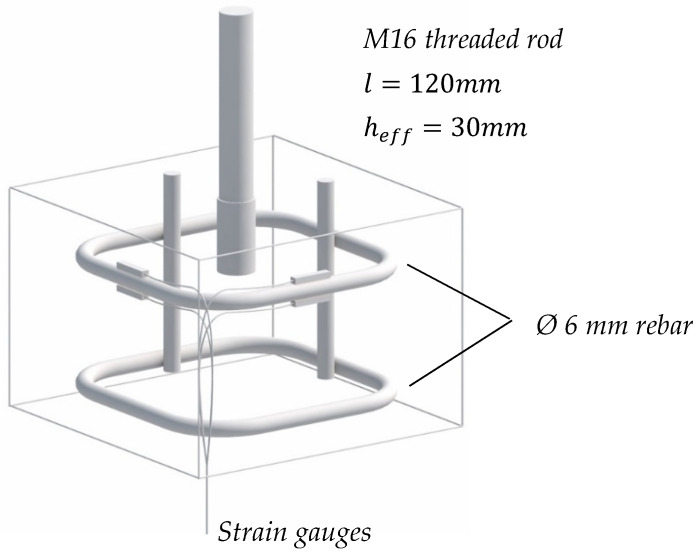
Radial stress measurement probe.

**Figure 12:**
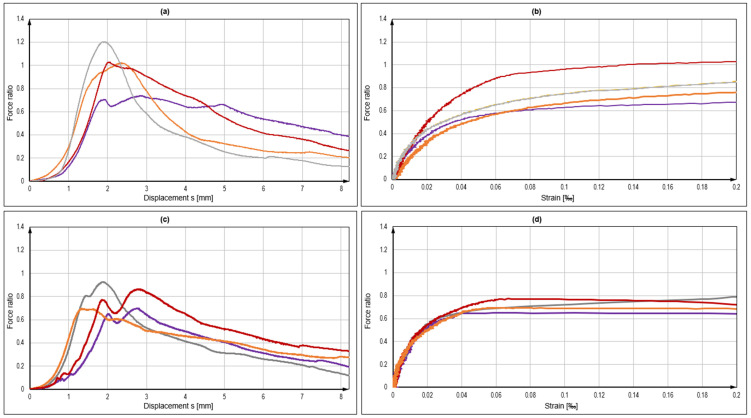
Load displacement curves of confined tests in SFRC (**a**) with corresponding strains (**b**) and of confined tests in plain concrete (**c**) with corresponding strains (**d**). Colours in the left graphs correspond to the ones in the right graphs, but they are otherwise randomly selected signify different samples in the same test series.

In order to evaluate the confinement of bonded anchors in relation to the borehole quality and addition of fibre reinforcements, an additional series of tests was carried out, particularly for product B. With the loading configuration remaining identical as described in the previous section, small-scale specimens were designed and used again under confined pull-out testing. In this case, the borehole depth was limited to 35 mm, allowing for an embedment depth of 30 mm. The specimens had a plan dimension of 150 by 150 mm, a thickness of 100 mm and at the mid-level of the embedment depth, i.e., at the 20 mm depth from the upper surface, they were equipped with a 6 mm confinement reinforcement stirrup. The specimens were produced by casting them in 150 mm cubes and cutting the upper 50 mm of the specimen, where the anchor would, subsequently, be placed in order to reduce the effects of a nonhomogeneous fibre distribution due to the casting boundary [51,52]. Strain gauges were attached in two directions of the stirrup to calculate the average exerted radial stress during the test as a comparison between plain and steel fibre-reinforced specimens. A sketch of the used specimen is provided in Figure 11, and the results are collectively presented in Figure 12.

**Figure 13:**
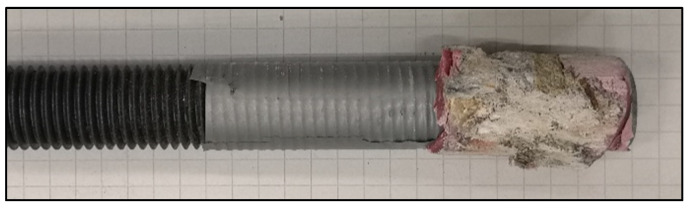
Typical pattern of failure in boreholes with bonded length of 30 mm.

As seen in Table 3, the loads achieved in SFRC were generally higher than plain concrete, although with a higher scatter as well. This confirmed the confinement and load increase in anchors in SFRC compared to anchors in plain concrete as mentioned above. Most importantly, the stress retention in the vicinity of anchors in SFRC was higher and it remained ascending for a tensile strain beyond approx. 0.1‰, which represented the failure strain of plain concrete in tension. This indicated that, although radial splitting cracks developed in both cases, SFRC could still provide a postcracking resistance and a higher robustness against the loss of adhesion. In contrast to tests with a full bond length, the failure plane was identified at both the concrete–mortar and the thread-mortar interfaces interchangeably (Figure 13). 

## 7. Conclusions

The present paper contributes to the current technical discourse of the influence of steel fibre reinforcement in concrete on the installation quality and load-bearing performance of post-installed bonded anchors. It initially presented background technical information and relevant studies, which indicated a knowledge gap in understanding the influence of steel fibres in the performance of bonded anchors in concrete. A multilateral assessment of this effect was presented here, while two main parameters were varied, i.e., the inclusion or not of high-strength fibres in concrete and the use of two different types of polymer-based bonding mortars. The assessment included a high-level and microscopic qualitative investigation of the borehole surface roughness, full-scale pull-out tests to assess the bond strength of the anchors, an FE simulation of the tests and an assessment of fibre contribution in the load-bearing and confinement of the anchors, and, finally, a small-scale test with a measurement of the confinement and splitting stress retention through fibres with strain gauge measurements.

The borehole roughness analyses indicated that the borehole roughness could be influenced by the presence of steel fibre reinforcement, mainly due to the protrusion of fibres in the borehole. This may be related to an increase in the bond strength of anchors in SFRC. Furthermore, this calls for a more insightful investigation of the drilling processes and drilling tool adjustments for the installation of anchors in SFRC.

The pull-out results suggested that a mild increase in the bond strength could be expected in SFRC when high-strength mortars were used, i.e., when the load-bearing of the anchor relied strongly on the concrete–mortar interface. A substandard or generally inadequate borehole cleaning prior to the installation of the bonded anchor led to a significantly reduced pull-out resistance. This quality difference was by far more relevant to the performance of the anchors than the inclusion of fibres in the concrete mix. A slightly varying load-bearing performance under pull-out was observed between the plain concrete and SFRC, but this may have also been attributed to the overall variability related to the low installation quality. For anchors prepared and installed per the recommended procedures, those for Product A did not show a strong influence of the anchor performance by steel fibres in the concrete mix, while failures occurred predominantly at the mortar–thread interface through the shearing-off of the mortar. Anchors installed by means of Product B exhibited an approx. 10% higher overall resistance in SFRC at the mean value level. At the characteristic value, a load increase of more than 10% in SFRC was also observed at the characteristic value levels for both Products A and B. Additionally, the levels of deformation at the loss-of-adhesion for bonded anchors in SFRC were found to be higher as compared to plain concrete.

The FE analyses indicated a contribution of fibres in the area around the bonded anchor, but this was of a rather inconsequential magnitude in regards to the load-bearing performance pull-out of the anchor. However, the confinement of the concrete close to the surface suggested that a possible influence could be present between the ratios of bond strengths measured by confined and unconfined test set-ups.

Small-scale tests also contributed to the conclusions above since both bond strengths and confinement strains were found to be higher in SFRC as compared to plain concrete.

In general, all studies converged to the conclusion that, for the tested configurations of materials and load set-ups, the resistance and robustness of bonded anchors in SFRC was not inferior to tests in equivalent plain concrete. This was also the indication in regards to the long-term performance of bonded anchors, since the deformation at the loss of adhesion was proportional to the qualification criterion of bonded anchors under sustained loads. The overall load–displacement performance of anchors in SFRC corresponded to typical patterns met in previous literature on bonded anchors in plain concrete. Moreover, some improvement was seen in terms of the displacement and load-bearing capacity, which was also in agreement with existing knowledge for bonded anchors in fibre-reinforced concrete.

Further investigations on finer quality influences such as the roughness of the internal borehole surface, the drilling method, as well as variations in the fastening products and concrete/SFRC mixes could allow an improved insight on possible influences of steel fibre reinforcement on the performance of bonded anchors in concrete. Moreover, the comparison of confined and unconfined tests could lead to a clearer understanding of the combined bond and breakout failure mode and, consequently, the load capacity dependence on both the borehole quality and the concrete homogeneity and quality close to the free surface of SFRC components. Finally, a significant potential in the design service life of bonded anchors could be disclosed for bonded anchors in SFRC in long-term loading tests.

## Figures and Tables

**Table 1:** Overview of pull-out tests.

Test ID	Bonding Mortar	Borehole Cleaning	Substrate Concrete	Sample Size (-)
A1	Product A	Yes	Plain	4
A1x	Product A	No	Plain	4
A2	Product A	Yes	SFRC	4
A2x	Product A	No	SFRC	4
B1	Product B	Yes	Plain	6
B1x	Product B	No	Plain	4
B2	Product B	Yes	SFRC	5 + 1 outlier
B2x	Product B	No	SFRC	4

**Table 2:** Overview of pull-out test results normalized by A1/B1 mean value as reference (standard installation in plain concrete).

Test ID	Mean Failure Load Normalized (-)	Coefficient of Variation (-)	Characteristic Resistance Normalized (-)	Mean Displacement at Peak Load (mm)	Mean Displacement at Loss of Adhesion (mm)
A1	1.00	0.091	1.00	1.6	1.6
A1x	0.68	0.089	0.68	1.8	1.8
A2	0.98	0.025	1.12	1.5	1.5
A2x	0.68	0.250	0.44	3.4	3.4
B1	1.00	0.075	1.00	2.7	2.7
B1x	0.44	0.074	0.44	8.5	8.5
B2	1.10	0.026	1.22	3.0	3.0
B2x	0.47	0.088	0.46	11.0	11.0

**Table 3:** Overview of pull-out test results normalized by H1 mean value as reference (standard installation in plain concrete).

Test ID	Substrate Concrete	Sample Size (-)	Mean Failure Load (-)	Coef. of Variation (-)	Mean Displacement (mm)
H1	Plain	4	1.00	0.146	2.3
H2	SFRC	4	1.25	0.193	2.3

## Data Availability

Not applicable.
